# Stifle kinematics in 4 dogs with cranial cruciate ligament insufficiency treated by CORA-based leveling osteotomy

**DOI:** 10.3389/fvets.2022.1052327

**Published:** 2022-12-02

**Authors:** Selena Tinga, Natalie Hughes, Stephen C. Jones, Brian Park, Lindsey Palm, Sasank S. Desaraju, Scott A. Banks, Sandra L. MacArthur, Daniel D. Lewis

**Affiliations:** ^1^Department of Veterinary Clinical Sciences, The Ohio State University College of Veterinary Medicine, Columbus, OH, United States; ^2^Department of Small Animal Sciences, College of Veterinary Medicine, University of Zurich, Zurich, Switzerland; ^3^Department of Mechanical and Aerospace Engineering, College of Engineering, University of Florida, Gainesville, FL, United States; ^4^Department of Small Animal Clinical Sciences, College of Veterinary Medicine, University of Florida, Gainesville, FL, United States

**Keywords:** cranial cruciate ligament, CORA, CBLO, kinematics, stifle

## Abstract

**Objective:**

The purpose of this study was to quantify three-dimensional (3D) stifle kinematics during walking in dogs with complete cranial cruciate ligament insufficiency (CCL-I) treated with a CORA-based leveling osteotomy (CBLO).

**Study design:**

Four client-owned dogs with unilateral complete CCL-I were prospectively enrolled. Custom digital 3D models of the femora and tibiae were created from pre-and postoperative computed tomographic scans for each dog. Lateral view fluoroscopic images were collected during treadmill walking preoperatively and 6 months after CBLO. Results were generated using a 3D-to-2D image registration process. Pre-and postoperative stifle kinematics (craniocaudal translation, extension angle) were compared to that of the unaffected contralateral (control) stifle. Force plate gait analysis was performed, and symmetry indices (SI) were calculated for peak vertical force (PVF) and vertical impulse (VI).

**Results:**

After CBLO, craniocaudal femorotibial motion was reduced by a median (range) of 43.0 (17.0–52.6) % over the complete gait cycle. Median (range) PVF SI was 0.49 (0.26–0.56) preoperatively and 0.92 (0.86–1.00) postoperatively, and VI SI was 0.44 (0.20–0.48) preoperatively and 0.92 (0.82–0.99) postoperatively.

**Conclusion:**

CBLO mitigated but did not fully resolve abnormal craniocaudal translation; lameness was substantially improved at 6 months.

## Introduction

Tibial plateau leveling osteotomy (TPLO) is a common procedure used to treat cranial cruciate ligament (CCL) insufficiency (CCL-I) in dogs. While the majority of dogs have good clinical outcomes after TPLO (1), TPLO does not completely restore normal stifle kinematics (2). In a fluoroscopic study (*n* = 16), 1/3 of dogs had persistent cranial tibial subluxation and instability and 2/3 of dogs developed stable caudal tibial subluxation post-TPLO (2). These kinematic alterations may influence the progression of stifle osteoarthritis and incite meniscal pathology (3), potentially affecting long-term comfort and function more than is currently appreciated. The center of rotation of angulation-based leveling osteotomy (CBLO) is an alternative tibial osteotomy procedure developed to address CCL-I in dogs (4–7). The CBLO aligns the proximal tibial anatomic and mechanical axes and has several purported advantages, such as minimizing caudal tibial subluxation (4–6). A cadaveric study reported that CBLO prevented cranial tibial translation after CCL transection throughout stifle range of motion, but no weight bearing force was applied (7). In addition, this study was *ex-vivo*; *in-vivo* kinematic outcomes have not been investigated after CBLO. This pilot study was intended to describe craniocaudal translation and extension angle of the stifle during walking in client-owned dogs treated with CBLO for naturally-occurring CCL-I.

## Materials and methods

The study was approved by the University's Institutional Animal Care and Use Committee, and owners provided informed consent. Client-owned dogs weighing 20–45 kg with unilateral lameness for up to 6 months were prospectively enrolled. Dogs had positive cranial drawer test and characteristic radiographic findings for CCL-I, and complete CCL rupture was confirmed arthroscopically. The CBLOs were performed by a board-certified surgeon experienced in the procedure. Dogs received cefazolin (30 mg/kg IV) every 90 min intraoperatively and every 8 h overnight, then a 10 to 14 day course of cephalexin (20–30 mg/kg orally twice daily) postoperatively. Intraoperative analgesia protocols varied according to attending clinician but included an epidural or peripheral nerve block, as well as systemic analgesics. Postoperative analgesia included non-steroidal anti-inflammatory drugs and a μ-agonist, followed by a course of oral non-steroidal anti-inflammatory drugs for at least 2 weeks. Surgical, anesthetic, and other perioperative care was not different from other clinical cases, except that 1.6 mm tantalum beads were implanted (4 to 6 beads into each femur and contralateral tibia and 3 to 4 beads into the affected tibia) to aid in kinematic measurements (8).

A detailed description of kinematic methods is available in previous reports (2, 8). Custom 3-dimensional (3D) digital models of the femora and tibiae, including metallic implants, were created from computed tomographic scans pre- and post-CBLO, and 3D coordinate systems were applied. Lateral fluoroscopy of the affected stifle was collected during treadmill walking preoperatively, and from the affected and contralateral (internal control) stifles at 6-months post-CBLO. Treadmill speed was set at 1 m/second and adjusted mildly until a natural walking cadence was achieved, if needed. Three gait cycles were analyzed and averaged for each set of fluoroscopic data. Stifle kinematics were measured by overlaying and precisely matching the 3D models to each fluoroscopic image. Custom software^1^,^2^ was used to determine the position of the femur and tibia in relation to each other, as the dog walked, as has been previously reported and validated (8).

*Craniocaudal position* (CCP) was defined as the craniocaudal distance between the femoral and tibial CCL attachments, and *cranial tibial translation* (CTT) was defined as a longer distance between femoral and tibial attachment sites in the affected stifle compared to contralateral stifle at a specific time point during the gait cycle. *Craniocaudal femorotibial* (CCFT) *motion* (the amount of cranial-caudal movement between the femur and tibia) was calculated by subtracting the minimum CCP from the maximum CCP over the gait cycle. Stifle extension angle was the measured offset between the long axes of the femur and tibia, with a larger value indicating stifle extension. Interobserver root mean square error (RMSE) was calculated for CCP and extension angle using 116 fluoroscopic frames (total frames from a complete gait cycle from each of 4 dogs; average of 29 frames per gait cycle).

Force plate gait analysis was performed pre- and 6 months post-CBLO. Dogs were walked across a force plate embedded in the floor until data from 3 successful trials were collected for each limb. Successful trials included those where only a single paw contacted the force plate at a time, there was minimal side-to-side head movement and no pulling on the leash, and walking velocity was within 10% of all other successful trials. Symmetry indices (SI) were calculated for peak vertical force (PVF) and vertical impulse (VI) from 3 trials using the formula: (9).


$$\begin{array}{l} \begin{array}{l} SI=1/3\sum_{k=1}^{3}\frac{affected}{contralateral}(1) \end{array} \end{array}$$

## Results

The four dogs enrolled completed the 6-month study; none of the dogs developed signs of contralateral CCL-I. Breeds included 3 Labrador Retrievers and 1 German Shorthaired Pointer. Median (range) age was 6.5 (2.2–7.6) years and weight was 34.5 (22.3–41.0) kg. Dogs were lame for a median (range) of 4.2 (2–6) months preoperatively. Three dogs had tearing of the caudal horn of the medial meniscus that was debrided, and one dog had no meniscal pathology and no meniscal treatment. Median (range) tibial plateau angles (TPA) were 25.8° (24–31) preoperatively and 8.5° (3–20) post-CBLO. Using 116 images (1 complete gait cycle from each of the 4 dogs), interobserver RMSE was 0.93 mm for CCP and 2.26° for extension angle.

### Craniocaudal translation

**Figure 1:**
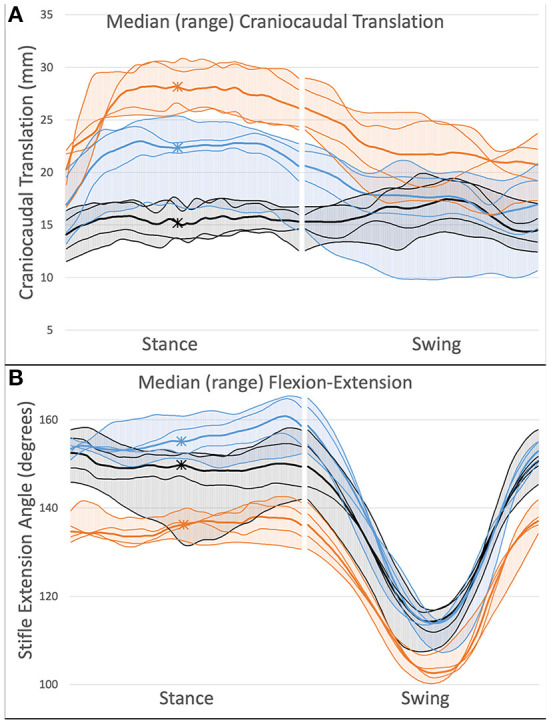
Kinematic outcomes after CBLO in 4 dogs. Increased y-value indicates cranial tibial translation **(A)** or stifle extension **(B)**. X-axis represents progression through the gait cycle with the break in data indicating the transition from stance to swing phase. Asterisks mark mid-stance phase. Orange line, Preoperative; Blue line, Post-CBLO; Black line, Control. Thick lines represent median (range) values and thin lines represent individual dog values. CBLO resulted in incomplete mitigation of cranial tibial translation **(A)** and increased extension angle **(B)** during stance phase.

At mid-stance phase, the affected stifle had median (range) CTT of 12.7 (12.0–14.6) mm preoperatively and 6.9 (3.1–9.2) mm post-CBLO (Figure 1A, Table 1). Post-CBLO, abnormal CCFT motion was reduced by a median (range) of 43.0 (17.0–52.6) %.

**Table 1:** Kinematic and kinetic results [median (range)].

		**Preoperative**	**6 months post–CBLO**	**Contralateral limb**
Craniocaudal femorotibial motion (mm)	Stance phase	9.5 (4.1–11.7)	5.0 (4.0–8.0)	2.0 (1.6–2.9)
	Swing phase	7.2	5.1	3.3
		(5.8–9.7)	(2.7–5.8)	(1.9–4.3)
	Complete gait cycle	9.9	7.8	3.6
		(7.6–11.7)	(5.1–8.4)	(2.9–4.3)
Extension angle ROM (degrees)	Stance phase	5.1 (3.3–9.9)	8.2 (5.1–12.1)	6.6 (4.7–14.4)
	Swing phase	34.0	45.0	39.4
		(30.6–40.3)	(40.5–57.7)	(33.2–45.9)
	Complete gait cycle	35.2	47.3	39.9
		(33.2–41.2)	(40.5–58.2)	(33.2–46.4)
Kinetic results (symmetry indices)	Peak vertical force	0.49 (0.26–0.56)	0.92 (0.86–1.00)	n/a
	Vertical impulse	0.43	0.92	n/a
		(0.20–0.48)	(0.82–0.99)	

Kinematic results include craniocaudal femorotibial motion and extension angle range of motion (ROM). Kinetic results include symmetry indices for peak vertical force and vertical impulse.

### Stifle extension angle

Preoperatively, all dogs had decreased stifle extension throughout the gait cycle (Figure 1B, Table 1). After CBLO, 3 dogs had increased stifle extension during stance phase and extension was not different from control during swing phase, while 1 dog had a stifle extension angle that was indistinguishable from control throughout the entire gait cycle.

### Kinetic gait analysis

Median (range) walking velocity during force plate gait analysis was 1.6 (1.3–1.9) m/second preoperatively and 1.9 (1.5–2.1) m/second 6-months post-CBLO. Force plate SIs improved and were symmetrical or near symmetrical for all dogs 6 months after CBLO (Table 1).

## Discussion

The primary objective of this pilot study was to report *in-vivo* CTT and stifle extension angle after CBLO in 4 dogs, in comparison to the preoperative CCL-I and contralateral normal stifles. Although limb function improved substantially in all dogs, CBLO mitigated, but did not eliminate abnormal craniocaudal motion in any dog, and 3 of 4 dogs developed hyperextension of the stifle during stance phase post-CBLO.

After CBLO, kinematic analysis identified residual CTT during stance phase and residual CCFT motion throughout the gait cycle in all 4 dogs compared to the contralateral stifle, though these were reduced by a median of 48.0 and 43.0%, respectively, when compared to pre-operative. In a previous study, 5/16 dogs had residual CTT and CCFT motion after TPLO, while 10/16 dogs developed *caudal* tibial subluxation (without abnormal CCFT motion) (2). After TPLO, the mean ± SD TPA in dogs with cranial tibial subluxation (7.2° ± 2.6) was significantly higher than in dogs with caudal tibial subluxation (2.9° ± 2.7) (2). While a TPA of 6.5° has been suggested as optimal after TPLO (10), a TPA of 9–12° has be recommended after CBLO, with this slightly higher TPA intended to lessen the potential for caudal tibial translation (4, 11). There was a wide range of post-CBLO TPAs: 2 dogs had a TPA <9°, 1 dog had a TPA of 10°, and 1 dog had a TPA of 20°. The cause for incomplete plateau leveling in the dog with the highest TPA was not identified upon debrief among the surgeons involved but could be avoided by use of surgical guides. The dog with the highest TPA had the least resolution of CTT and CCFT motion, but CBLO did not normalize stifle kinematics in any dog.

Importantly, all dogs regained symmetrical or near-symmetrical weight-bearing by 6 months post-CBLO with median SIs of 0.92 for both PVF (range: 0.86–1.00) and VI (range: 0.82–0.99), despite residual kinematic abnormalities. The median hind limb SIs of 0.92 is similar to that reported at 6 months post-TPLO (12). Improved gait symmetry, plus normal to increased stifle extension angle, is likely supportive of improved stifle comfort after CBLO.

This near-symmetrical weight-bearing, however, is unlikely to represent near-normal joint health. A study reporting second look arthroscopy findings in 35 dogs with complete or incompetent partial CCL-I found that 13/35 dogs had new cartilage lesions (modified Outerbridge score of 1 or 2) on at least 1 cartilage surface at a median of 12 months post-CBLO (6). Furthermore, of the 16 dogs with intact menisci and no meniscal treatment performed during CBLO, late onset medial (*n* = 7) or lateral (*n* = 3) meniscal lesions were noted in 10 dogs at the time of second look arthroscopy (6). Results of second-look arthroscopic evaluations have also been reported after TPLO, where 39/46 stifles with complete or incompetent partial CCL-I were reported to have cartilage lesions not visible at the original surgery, at a median of 35.5 months post-TPLO (13). Additionally, of the 27 dogs with intact menisci and no meniscal treatment performed during TPLO, 10 had new meniscal pathology at second-look (13). While these studies are not directly comparable, particularly due to the extreme difference in follow up time, we suggest that persistent kinematic abnormalities was likely present in some CBLO- and TPLO-treated stifles in these studies and may have contributed to the progression of articular cartilage lesions and meniscal pathology that was reported (6, 13).

This study has substantial limitations that prevent the authors and readers from proclaiming generalizations regarding the effects of CBLO on stifle kinematics, with the principal limitation being the small sample size. Post-CBLO TPAs varied widely, including one dog having a post-CBLO TPA higher than the recommended range; post-CBLO TPAs did not appear to have a direct influence on the magnitude of persistent stifle instability in these dogs. Other osseus morphologic outcomes may explain this kinematic outcome, but varus/valgus and torsional morphology were not evaluated. Kinematic data collection was performed during treadmill walking, which likely caused mild changes to joint kinematics (14). Lastly, we used 6-month post-CBLO data from each dog's contralateral stifle for control data, and although we screened for clinical evidence of other musculoskeletal disease, we cannot exclude the possibility of early contralateral CCL-I or other disease. The fluoroscopic method used here for determination of stifle kinematics has been validated for canine stifles by comparison to biplanar radiostereometric analysis and, using 25 images from 5 simulated gait cycles from 1 cadaver, this methodology was found to have a RMSE between techniques of 0.39 mm for cranial tibial translation and 0.67° for extension angle (8). However, strong kinematic methodology cannot completely overcome the discussed limitations of low case number and high variability.

All four dogs in this pilot study showed marked improvement in limb function 6 months post-CBLO with decreased but persistently abnormal CCFT motion during gait. A larger prospective study is warranted to determine the incidence and severity of abnormal kinematics after CBLO. Future studies should also be designed to attempt to identify the cause(s) of postoperative kinematic abnormalities including proximal tibial morphology; results of such a study could refine planning and execution of the procedure if risk factors for poor outcome can be identified. In addition, whether stifle kinematics normalize with longer follow-up and/or improved muscle mass, and what the consequences of the presence and severity of kinematic abnormalities are on joint health, should be evaluated.

## Data availability statement

The raw data supporting the conclusions of this article will be made available by the authors, without undue reservation.

## Ethics statement

The animal study was reviewed and approved by Institutional Animal Care and Use Committee at the University of Florida. Written informed consent was obtained from the owners for the participation of their animals in this study.

## Author contributions

ST oversaw the majority of the project, specifically study design, data analysis, and writing. NH performed data analysis and writing, under the supervision of ST. SJ assisted with study design, data analysis, and writing. LP, BP, SD, and SB provided engineering and software support. SM was primarily responsible for data collection under the supervision of DL. DL assisted with study design, case enrollment, surgical treatment, data analysis, and writing. All authors contributed to the article and approved the submitted version.

## Funding

Supported in part by Veterinary Orthopedic Implants and discretionary funding from the University of Florida's Comparative Orthopedic and Biomechanical Laboratory.

## Conflict of interest

The authors declare that the research was conducted in the absence of any commercial or financial relationships that could be construed as a potential conflict of interest.

## Publisher's note

All claims expressed in this article are solely those of the authors and do not necessarily represent those of their affiliated organizations, or those of the publisher, the editors and the reviewers. Any product that may be evaluated in this article, or claim that may be made by its manufacturer, is not guaranteed or endorsed by the publisher.

## Footnotes

^1^JointTrack, University of Florida: http://sourceforge.net/projects/jointtrack/.

^2^MATLAB and Statistics Toolbox Release R2015a, The MathWorks, Inc., Natick, MA.
